# Senescent Fibroblasts Drive FAP/OLN Imbalance Through mTOR Signaling to Exacerbate Inflammation and Bone Resorption in Periodontitis

**DOI:** 10.1002/advs.202409398

**Published:** 2024-12-24

**Authors:** Chenghu Yin, Liangliang Fu, Shuling Guo, Youde Liang, Taizhi Shu, Wenjun Shao, Haibin Xia, Ting Xia, Min Wang

**Affiliations:** ^1^ State Key Laboratory of Oral & Maxillofacial Reconstruction and Regeneration Key Laboratory of Oral Biomedicine Ministry of Education Hubei Key Laboratory of Stomatology School & Hospital of Stomatology Wuhan University Wuhan 430079 China; ^2^ Department of Stomatology Center The People's Hospital of Baoan Shenzhen (The Second Affiliated Hospital of Shenzhen University) Shenzhen Guangdong 518081 P. R. China

**Keywords:** cellular senescence, fibroblast activation protein, macrophage, osteolectin, periodontitis

## Abstract

Fibroblast activation protein (FAP), predominantly expressed in activated fibroblasts, plays a key role in inflammatory bone diseases, but its role in periodontitis remains unclear. Accordingly, this study identified a positive association between FAP levels and periodontitis susceptibility using Mendelian randomization analysis. Human and mouse periodontitis tissues show elevated FAP and reduced osteolectin (OLN), an endogenous FAP inhibitor, indicating a FAP/OLN imbalance. Single‐cell RNA sequencing revealed gingival fibroblasts (GFs) as the primary FAP and OLN source, with periodontitis‐associated GFs showing increased reactive oxygen species, cellular senescence, and mTOR pathway activation. Rapamycin treatment restored the FAP/OLN balance in GFs. Recombinant FAP increased pro‐inflammatory cytokine secretion and osteoclast differentiation in macrophages, exacerbating periodontal damage, whereas FAP inhibition reduced macrophage inflammation, collagen degradation, and bone resorption in experimental periodontitis. Therefore, senescent fibroblasts drive the FAP/OLN imbalance through mTOR activation, contributing to periodontitis progression. Consequently, targeting FAP may offer a promising therapeutic strategy for periodontitis.

## Graphical abstract

The imbalance of fibroblast activation protein (FAP) and osteolection (OLN) in fibroblasts promotes the progression of periodontitis. Periodontitis causes excessive activation of the mTOR signaling pathway in fibroblasts, accelerating cellular senescence. Senescent fibroblasts are featured by overexpression of FAP and decreased expression of OLN, which promote macrophages to obtain pro‐inflammatory and osteoclast phenotypes, thereby exacerbating bone resorption in periodontitis.

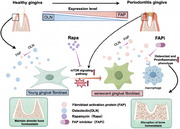

## 1 Introduction

Periodontitis is a chronic inflammatory disease characterized by clinical attachment loss and alveolar bone destruction. It causes tooth displacement, migration, and excessive mobility, eventually leading to tooth loss and compromising masticatory function.^[^ ^1^, ^2^ ^]^ Periodontitis remains the leading cause of adult tooth loss worldwide, significantly impacting the quality of life and imposing a substantial socioeconomic burden and healthcare costs globally.^[^ ^3^ ^]^

Bacterial biofilms represent the primary etiological factor of periodontitis, while an excessive host immune inflammatory response plays a pivotal role in periodontal tissue destruction.^[^ ^4^ ^]^ The routine treatment for periodontitis currently includes subgingival debridement and scaling and root planing (SRP) to remove subgingival biofilms and calculi.^[^ ^5^ ^]^ However, these measures often cannot fully address the clinical needs, particularly for difficult‐to‐reach sites and patients unresponsive to non‐surgical periodontal therapy, often due to persistently dysregulated immune inflammatory responses.^[^ ^6^ ^]^ Consequently, there is a spreading trend toward exploring adjunctive therapies aimed at modulating the host immune response to enhance and optimize SRP outcomes.

Fibroblast activation protein (FAP) might be a promising immunity‐regulating target in bone inflammatory diseases. FAP belongs to the serine protease family with dipeptidyl peptidase and endopeptidase activities, sharing structural and functional similarities with dipeptidyl peptidase IV (DPPIV).^[^ ^7^, ^8^ ^]^ Elevated DPPIV expression and activity are associated with the severity of periodontitis.^[^ ^9^ ^]^ DPPIV inhibitors (such as sitagliptin) can reduce inflammation in bone diseases, although with significant sex‐dependent effects.^[^ ^10^, ^11^ ^]^ FAP is typically expressed in activated fibroblasts, being extensively involved in the formation of a tumor microenvironment, inflammation, and wound healing.^[^ ^12^, ^13^ ^]^ In musculoskeletal diseases, FAP has been identified as a critical player in the destruction of hard tissues in rheumatoid arthritis and osteoarthritis.^[^ ^14^, ^15^ ^]^ Unlike sterile bone inflammation (e.g., osteoarthritis), periodontitis involves a complex interplay between bacterial infection and host immune responses, destructing soft and hard periodontal tissue to varying degrees.^[^ ^4^ ^]^ In oral health, FAPα regulates osteoclast differentiation during the development of chronic apical periodontitis.^[^ ^16^ ^]^ Notably, FAP signals significantly increased in the gingival tissues of periodontitis patients, as assessed by positron emission tomography.^[^ ^17^ ^]^ Thus, FAP might be involved in the progression of chronic periodontitis. However, the role and mechanism of FAP in periodontitis remain unexplored.

Osteolectin (OLN), an endogenous FAP inhibitor, can interact with FAP and inhibit its protease activity.^[^ ^8^, ^14^, ^18^ ^]^ The balance between OLN and FAP controls bone homeostasis and the immune microenvironment. Studies showed aging can disrupt this balance, leading to osteoporosis characterized by significantly increased FAP activity and decreased OLN levels.^[^ ^8^, ^19^ ^]^ Additionally, a senescent pathogenic cell cluster in osteoarthritis features FAP dysfunction, whereas FAP overexpression promotes chondrocyte senescence and the senescence‐associated secretory phenotype (SASP).^[^ ^20^ ^]^ Hence, cellular senescence might play a role in regulating FAP function.^[^ ^21^ ^]^ However, the underlying mechanism by which cellular senescence influences the balance between FAP and OLN needs further elucidation.

This study found a disruption in the balance between FAP and OLN in periodontitis, manifested by increased FAP and decreased OLN expression. FAP exacerbates periodontal destruction through direct collagenase activity and indirect regulation of the macrophage phenotype. Mechanistically, the senescence of gingival fibroblasts, accompanied by the activation of the mTOR pathway, plays a crucial role in disrupting the FAP and OLN balance. Finally, using a selective FAP small‐molecule inhibitor in vivo successfully alleviated periodontitis progression. These results elucidate the mechanisms by which cellular senescence regulates the balance between FAP and OLN and demonstrate that FAP could be a potential therapeutic target for periodontitis.

## 2 Results

### 2.1 The Balance of FAP and OLN is Disrupted During Periodontitis Progression

**Figure 1:**
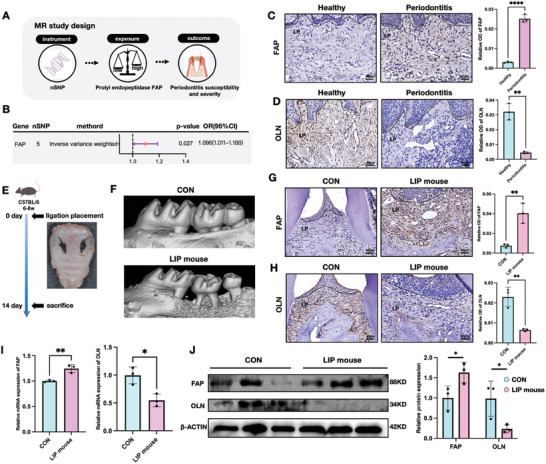
FAP/OLN balance was interrupted in the human gingiva and mouse model of the periodontitis. A). Study design of Mendelian randomization (MR). B) MR results of the validation phase, which revealed that elevated FAP levels were significantly associated with periodontitis. C,D) Representative IHC staining and MOD values of FAP and OLN in healthy and periodontitis patient gingiva. A significant increase of FAP expression and a notable decrease of OLN expression within the gingival connective tissue (lamina propria) of periodontitis patients compared with healthy gingiva. Scale bar: 40 µm; LP: lamina propria. E) Strategy of ligature‐induced periodontitis (LIP) mouse model. F) Micro‐CT images and 3‐D visualization of the maxilla. G,H) Representative IHC staining and MOD values of FAP and OLN in control wild and Ligature induced periodontitis (LIP) mouse model. Elevated FAP expression and reduced OLN expression were found in LIP mouse model than the control model. Scale bar: 40 µm. I) RT‐PCR analysis quantified relative gene expression of Fap and Oln normalized to β‐actin. J) Western blot images and semi‐quantification of FAP and OLN protein levels in control and LIP mouse gingiva. Values are shown as the means±SD; *n* = 3; ^*^ *p* < 0.05; ^**^ *p* < 0.01; ^***^ *p* < 0.001; and ^****^ *p* < 0.0001; MOD: mean optical density.

We utilized the latest genome‐wide association study (GWAS) data to conduct Mendelian randomization (MR) analysis (**Figure** **1**A) to explore the potential causal relationship between FAP and periodontitis.^[^ ^22^ ^]^ As 5 single nucleotide polymorphisms (SNPs) were selected as instrumental variables for FAP levels, the inverse variance weighting (IVW) analysis revealed that elevated FAP levels were significantly associated with periodontitis (odds ratio = 1.096, 95% confidence interval = 1.011–1.188, *p* = 0.027) (Figure 1B). No significant evidence of heterogeneity or pleiotropy was detected (*p* > 0.05) (Tables S2 and S3, Supporting Information). Additionally, leave‐one‐out sensitivity tests demonstrated the stability of the MR findings (Figure S1, Supporting Information). The complex of FAP with its endogenous inhibitor OLN, which impacts the outcomes of bone diseases such as osteoarthritis, was predicted by AlphaFold3 (Figure S2, Supporting Information) and validated through co‐immunoprecipitation assays.^[^ ^14^ ^]^ Subsequently, we assessed the expression of FAP and its endogenous inhibitor OLN in gingival tissue specimens from healthy individuals and patients with periodontitis. Immunohistochemical analysis indicated a significant increase in FAP expression within the gingival connective tissue (lamina propria) of patients with periodontitis (Figure 1C), accompanied by significantly decreased OLN expression (Figure 1D). These observations were further validated using a ligature‐induced periodontitis (LIP) mouse model (Figure 1E,F), which similarly exhibited elevated FAP (Figure 1G) and reduced OLN expression (Figure 1H) in gingival tissues. Quantitative real‐time polymerase chain reaction (PCR) and Western blot analyses also supported these findings, showing a significant upregulation of FAP and downregulation of OLN in gingival tissues from LIP mice compared to control mice (Figure 1I,J). Therefore, the balance between FAP and OLN is disrupted during periodontitis progression. This disruption underscores the potential role of FAP dysregulation in periodontal tissue damage, highlighting its importance as a potential therapeutic target.

### 2.2 The FAP/OLN Balance is Restored with Periodontitis Regression, with Gingival Fibroblasts Serving as the Main Source of FAP and OLN

**Figure 2:**
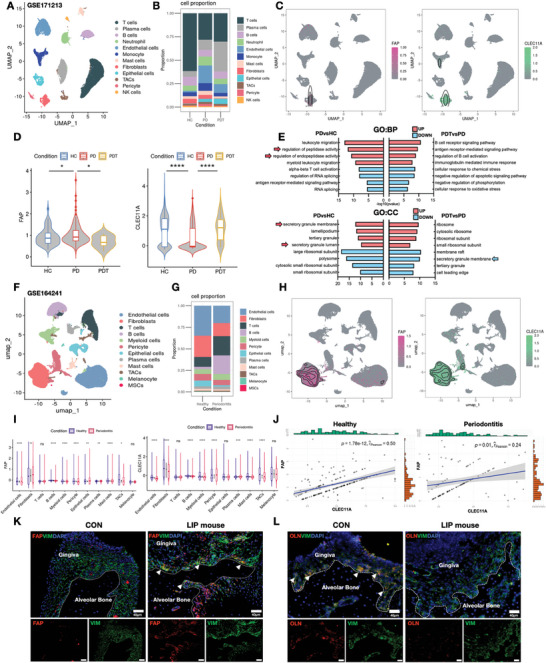
FAP/OLN balance is restored with periodontitis regression and gingival fibroblasts are the main source of FAP and OLN. A) UMAP diagram of single‐cell annotation for healthy control (HC), periodontitis (PD), and periodontitis after treatment (PDT) patients from sc‐RNA data GSE171213, which included 12 identified cell types. B) Histogram of gingival tissue cell ratio in HC, PD, and PDT patients. C) FAP and CLEC11A (encoding OLN gene) were mainly expressed in the fibroblasts cluster. D) Expression levels of FAP and CLEC11A in fibroblasts with HC, PD, and PDT patients, which suggested that FAP and OLN expression were disrupted in periodontitis and could be reversed after inflammation resolution. E) Gene Ontology of biological process and cellular component with differentially expressed genes in fibroblasts in PD versus HC or PDT versus PD, which indicated significant upregulation of inflammation‐related pathways as well as the endopeptidase pathway in periodontitis fibroblasts and increased enrichment of secreted proteins in periodontitis fibroblasts labeled by the red arrow while notably decreased following periodontal therapy indicated by the blue arrow. F) UMAP diagram of single‐cell annotation for the healthy and periodontitis samples from sc‐RNA data GSE164241. G) Histogram of gingival tissue cell ratio in healthy and periodontitis patients. H) FAP and CLEC11A (encoding OLN gene) were also mainly expressed in the fibroblasts cluster. I) The violin plot showing expression levels of FAP and CLEC11A in each cell type with healthy and periodontitis patients, which suggested that higher expression of FAP and lower expression of CLEC11A in periodontitis fibroblasts than that in healthy fibroblasts inconsistent with results from Figure 2D. J) Correlation analysis showing expression correlations of FAP with OLN in the healthy and periodontitis gingiva fibroblasts, which suggested a disrupted co‐expression pattern in periodontitis. K,L) IF staining of FAP or OLN (red), VIM (green, a typical fibroblast marker), and nuclei (blue) in control and LIP mouse gingiva, which suggested that FAP^+^ fibroblasts rather than OLN^+^ fibroblasts predominated in the gingiva of periodontitis mice; white arrows indicate double positive cells. Scale bar: 40 µm.Values are shown as the means±SD; ^*^ *p* < 0.05; ^**^ *p* < 0.01; ^***^ *p* < 0.001; and ^****^ *p* < 0.0001.

We analyzed single‐cell RNA sequencing (scRNA‐seq) data GSE171213, which included 4 healthy control (HC) individuals, 5 periodontitis (PD) individuals, and 3 individuals with periodontitis after treatment (PDT), to further investigate the relationship between FAP, OLN, and periodontitis outcomes.^[^ ^23^ ^]^ Following data filtering and dimensionality reduction, gingival tissue cells were categorized into 18 distinct clusters (Figure S3A, Supporting Information), each defined by specific cell markers (Figure S3B, Supporting Information). These clusters were further grouped into 12 identified cell types (**Figure** **2**A,B). Expression analysis revealed that FAP and CLEC11A (encoding *OLN*) were predominantly expressed in the fibroblast cluster (Figure 2C). FAP and OLN expressions were significantly elevated and reduced, respectively, in fibroblasts from periodontitis patients. Interestingly, this imbalance was reversed following periodontal therapy (Figure 2D). We performed a Gene Ontology (GO) enrichment analysis on differentially expressed genes between healthy and periodontitis fibroblasts to explore the molecular mechanisms underlying the aforementioned changes. The analysis revealed significant upregulation of inflammation‐related and endopeptidase pathways in periodontitis fibroblasts. Furthermore, we observed increased enrichment of secreted proteins in periodontitis fibroblasts (indicated by the red arrow), which notably decreased following periodontal therapy (indicated by the blue arrow) (Figure 2E). Therefore, FAP with endopeptidase activity may be involved in periodontitis progression.

We analyzed single‐cell RNA sequencing data from a larger cohort, including 13 healthy and 8 periodontitis gingival samples, to further investigate the cellular sources and expression dynamics of FAP and OLN in gingival tissues.^[^ ^24^ ^]^ The analysis identified 16 distinct cell clusters defined by specific cell markers (Figure S3C,D, Supporting Information), which were categorized into 12 major cell groups (Figure 2F). Epithelial, endothelial, and immune cells, as well as fibroblasts, were the predominant cell types in the gingival tissue. In gingival tissues with periodontitis, the proportion of immune cell types, including T, B, myeloid, plasma, and mast cells, increased significantly, whereas other cell types, such as fibroblasts and endothelial cells, decreased (Figure 2G). Further analysis confirmed that FAP and CLEC11A (*OLN*) were predominantly expressed in fibroblasts (Figure 2H). FAP and CLEC11A expression changes in fibroblasts during periodontitis followed the same trends observed in Figure 2D (Figure 2I). Notably, the correlation coefficient (R^2^) between FAP and OLN expression in fibroblasts decreased from 0.5 in healthy fibroblasts to 0.24 in periodontitis fibroblasts (Figure 2J), suggesting a disrupted co‐expression pattern. Then, we examined FAP and OLN colocalization with vimentin (VIM), a fibroblast marker, in the gingival tissues of LIP mice using immunofluorescence for validation. The number of FAP^+^VIM^+^ double‐positive cells significantly increased in the gingival tissue of LIP mice compared to wild‐type controls, with these cells concentrated near the alveolar bone (Figure 2K; white arrow). Conversely, OLN^+^VIM^+^ double‐positive cells were abundant near the alveolar bone region in control mice but were scarcely distributed in the gingiva of periodontitis mice (Figure 2L; white arrow). Then, we stimulated primary human gingival fibroblasts in vitro with lipopolysaccharides from *Porphyromonas gingivalis* (Pg‐LPS). This stimulation caused a time‐dependent increase in FAP mRNA expression and a significant decrease in OLN mRNA expression (Figure S3E,F, Supporting Information). Thus, fibroblasts serve as the primary source of FAP and OLN in gingival tissues. Furthermore, their expression is modulated by the inflammatory microenvironment, particularly during periodontitis.

### 2.3 Cell Senescence Contributes to the Imbalance of FAP/OLN Expression in Gingival Fibroblasts in Periodontitis

**Figure 3:**
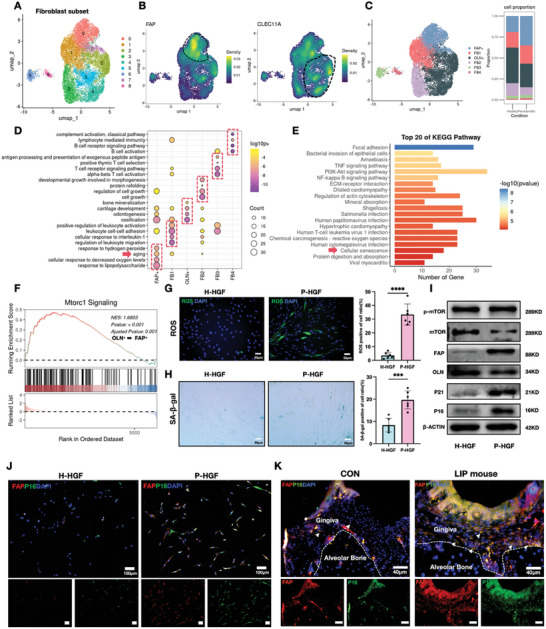
Cell senescence contributes to the imbalance of FAP/OLN expression in gingival fibroblasts in periodontitis. A) UMAP diagram illustrates the cell sub‐clusters of fibroblasts from GSE164241. B) Density map of FAP and CLEC11A (encoding OLN gene) expression in the UMAP diagram. C) UMAP diagram of single‐cell annotation of fibroblasts subsets and histogram of ratio in healthy and periodontitis patients. D) GO enrichment analysis of fibroblasts subclusters. The red arrow indicates the aging process is enriched in FAP^+^ fibroblasts. E) The top 20 of KEGG enrichment analysis in FAP^+^ fibroblasts. The red arrow indicates the cellular senescence process is enriched in FAP^+^ fibroblasts. F) GSEA enrichment analysis suggested that mTOR signaling pathway is enriched in FAP^+^ fibroblasts compared to OLN^+^ fibroblasts. G) ROS activity of H‐HGF and P‐HGF from human samples was observed using DCFH‐DA fluorescent probe, which indicated that P‐HGF exhibited higher level of ROS activity compared with H‐HGF. Green: ROS. Scale bar: 50 µm. H) Staining and quantitative analysis of SA‐β‐gal in H‐HGF and P‐HGF. Scale bar: 50 µm. I) Western blot showing cell senescence specific markers, p‐MTOR, MTOR, P16, P21, FAP, and OLN in H‐HGF and P‐HGF. J) IF staining of FAP (red), P16(green), and nuclei (blue) in H‐HGF and P‐HGF, which suggested that higher FAP and P16 proteins were in P‐HGF rather than H‐HGF. Scale bar: 100 µm. K) IF staining of FAP (red), P16 (green), and nuclei (blue) in control and LIP mouse gingiva, which suggested that more FAP and P16 double‐positive cells gathered in LIP mouse gingiva; white arrows indicate double‐positive cells. Scale bar = 40 µm.Values are shown as the means ± SD; ^*^ *p* < 0.05, ^**^ *p* < 0.01, ^***^ *p* < 0.001, and ^****^ *p* < 0.0001. H‐HGF: gingival fibroblasts from healthy gingiva; P‐HGF: gingival fibroblasts from periodontitis patient's gingiva.

We extracted fibroblast subpopulations and divided them into 9 distinct subgroups to further analyze the relationship between FAP and OLN expression changes in periodontal fibroblasts and their cellular functions (**Figure** **3**A). By examining FAP and OLN expression patterns across these subgroups, we found that subgroups 0 and 7 exhibited high FAP expression, whereas subgroups 2, 5, and 4 showed elevated OLN expression (Figure 3B). Following the reclassification of these subgroups and analysis of their proportional changes, we observed a significant increase in FAP^+^ fibroblasts and a decrease in OLN^+^ fibroblasts in periodontitis samples (Figure 3C). Afterward, we conducted GO and KEGG enrichment analyses on the top 300 highly expressed genes in each subgroup. GO enrichment revealed that FAP^+^ fibroblasts were associated with pathways linked to lipopolysaccharide response, hydrogen peroxide (H_2_O_2_) response, cellular hypoxia, and senescence. Conversely, OLN^+^ fibroblasts demonstrated potential for multidirectional differentiation, including pathways involved in osteogenesis, dentin formation, and chondrogenesis (Figure 3D). Notably, KEGG pathway analysis of the top 20 enriched pathways in FAP^+^ fibroblasts also highlighted the activation of the cellular senescence pathway (Figure 3E). Hence, the increased proportion of FAP^+^ fibroblasts in periodontitis was strongly associated with oxidative stress–induced cellular senescence. We performed GSEA enrichment analysis on the differentially expressed genes to identify the key pathways driving the transition between active OLN^+^ fibroblasts and senescence‐associated FAP^+^ fibroblasts. The activation of the MTORC1 pathway within the mTOR signaling pathway was a critical driver of this transition (Figure 3F). Elevated mTOR increases intracellular reactive oxygen species (ROS), ultimately causing DNA damage, inflammation, and accelerated cellular senescence,^[^ ^25^ ^]^ with mTORC1 serving as a critical regulatory node.^[^ ^26^ ^]^ This further underscores the pivotal role of senescence in the transition of fibroblasts toward a FAP^+^ fibroblast phenotype in periodontitis. Then, we evaluated senescence‐associated markers in healthy human gingival fibroblasts (H‐HGFs) and periodontitis human gingival fibroblasts (P‐HGFs) cultured in vitro. P‐HGF exhibited a higher proportion of ROS‐positive cells (Figure 3G) and an increased percentage of SA‐β‐gal‐positive cells (Figure 3H). Western blot analysis further revealed that the disruption of the FAP/OLN balance in P‐HGFs was accompanied by the upregulation of senescence‐related markers P16 and P21 and activation of the mTOR pathway, which is required for the maintenance or implementation of cellular senescence (Figure 3I). Immunofluorescence analysis demonstrated strong spatial colocalization of FAP and P16 in HGFs, with both proteins showing significantly elevated expression in P‐HGFs (Figure 3J). Similarly, we observed prominent colocalization of FAP and P16 in gingival tissues of a LIP mouse model compared to control mice (Figure 3K; white arrows). In conclusion, cellular senescence in gingival fibroblasts during periodontitis progression may play a critical role in the disruption of the balance of FAP and OLN expression.

### 2.4 Inhibiting the Cell Senescence of Fibroblast Rebalances the Expression of FAP and OLN

**Figure 4:**
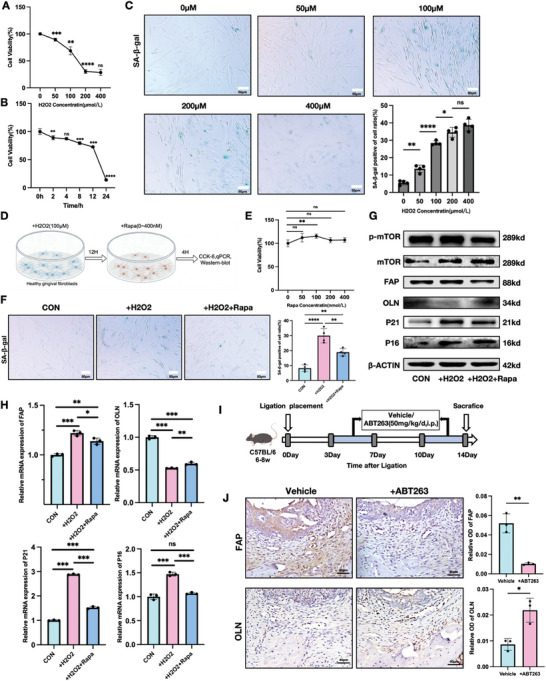
Inhibiting the cell senescence of fibroblast rebalances the expression of FAP and OLN. A) Cell viability detected by the CCK‐8 assay at different concentrations of H_2_O_2_ after 12 h of stimulation; *n* = 4. B) Cell viability of H‐HGF at concentration of 100 µm H_2_O_2_ for different stimulation time; *n* = 4. C) Staining and quantitative analysis of SA‐β‐gal of H‐HGF stimulated by different concentrations of H_2_O_2_. Scale bar = 50 µm. D) Experimental design of the following imageE‐H. H‐HGF was stimulated with 100 µm H_2_O_2_ for 12 h, and continued to culture for 12 h, then added different concentrations of Rapamycin (Rapa) for 4 h. E) Cell viability at different concentrations of Rapa after 4 h of stimulation; *n* = 4. F) Staining and quantitative analysis of SA‐β‐gal of H_2_O_2_‐stimulated H‐HGF with or without Rapa. Scale bar = 50 µm. G) Western blot image of p‐MTOR, MTOR, p16, P21, FAP, and OLN protein levels of H_2_O_2_‐stimulated H‐HGF with or without Rapa. H) RT‐PCR was used to quantify the relative gene expression levels of P16, P21, FAP, and OLN normalized to β‐ACTIN in H_2_O_2_‐stimulated H‐HGF with or without Rapa. I) The strategy of establishment of the mouse model of periodontitis treated with senolytic drug ABT263. J) Representative IHC staining and MOD values of FAP and OLN in vehicle and treatment with ABT263 groups, which suggested senolytic therapy could alleviate FAP/OLN imbalance in periodontits; *n* = 3; scale bar: 40 µm. Values are shown as the means±SD; ^*^ *p* < 0.05, ^**^ *p* < 0.01, ^***^ *p* < 0.001, and ^****^ *p* < 0.0001.MOD: mean optical density.

We first established an in vitro senescence model using HGFs by inhibiting DNA replication with the classical senescence‐inducing drug bleomycin (BLM) to validate the role of cellular senescence in the imbalance between FAP and OLN expression.^[^ ^27^ ^]^ BLM induced senescence in HGFs in a concentration‐dependent manner (Figure S4A, Supporting Information). Based on optimization experiments, we selected 50 µm BLM for 24 h for the optimal stimulation, which provided a significant increase in SA‐β‐gal expression (Figure S4B, Supporting Information). BLM stimulation increased FAP and decreased OLN expression in HGFs (Figure S4C,D, Supporting Information). To further simulate oxidative stress–induced ROS elevation and subsequent cellular senescence observed in periodontitis, while aligning with the activation of the related pathways identified in FAP^+^ fibroblasts from our previous findings, we opted to induce senescence in H‐HGFs by H_2_O_2_ treatment.^[^ ^28^ ^]^ H‐HGFs were exposed to varying concentrations of H_2_O_2_ (0–400 µm) for 12 h and 100 µm H_2_O_2_ for different durations (0–24 h) to determine the optimal concentration and exposure time for H_2_O_2_‐induced senescence. The optimal dose and duration were 100 µm H_2_O_2_ for 12 h: before this point, cell proliferation showed a steady, dose/time‐dependent inhibition, and SA‐β‐gal‐positive cells increased progressively (**Figure** **4**A–C). Exceeding these concentrations or exposure durations sharply decreased cell viability, causing significant morphological changes and cell death. Therefore, we selected 100 µm H_2_O_2_ for 12 h as the optimal condition for subsequent experiments.

We treated H_2_O_2_‐induced senescent HGFs with the classic anti‐senescence drug rapamycin (Rapa), a well‐established mTOR pathway inhibitor, to evaluate whether inhibiting cellular senescence could restore the FAP/OLN expression balance.^[^ ^29^ ^]^ After H_2_O_2_ exposure, the cells were allowed to recover under normal culture conditions for 12 h. Subsequently, cells in the experimental group were treated with Rapa, while the control group was maintained in a standard culture medium for 4 h (Figure 4D). The optimal Rapa concentration was 100 nm (Figure 4E). Under this condition, Rapa significantly reduced the number of SA‐β‐gal‐positive cells in HGFs compared to controls (Figure 4F). Western blot analysis revealed that Rapa reversed the H_2_O_2_‐induced upregulation of senescence‐associated proteins and restored the FAP/OLN protein balance. Additionally, Rapa inhibited the activation of the mTOR pathway (Figure 4G). Furthermore, qPCR analysis confirmed the beneficial effects of Rapa at the mRNA level (Figure 4H). Afterward, we administered the classical senolytic drug ABT263 to LIP mice to investigate whether senolytic therapy could alleviate FAP/OLN imbalance in vivo (Figure 4I).^[^ ^30^ ^]^ Immunohistochemical analysis showed that ABT263 treatment significantly improved the FAP/OLN imbalance in the gingival tissues of LIP mice compared to controls (Figure 4J). These findings demonstrate the critical role of cellular senescence in driving FAP and OLN imbalance, suggesting that targeting fibroblast senescence can effectively restore the balance between FAP and OLN expression.

### 2.5 Periodontal Injection of FAP Inhibitor Alleviates Periodontal Tissue Damage in Murine Periodontitis

FAP is a serine protease that promotes disease progression by regulating extracellular matrix degradation and inflammation.^[^ ^31^ ^]^ We performed bilateral periodontal local injections of FAP‐selective small‐molecule inhibitors (FAPi, 40 µg kg^−1^) or vehicle controls (DMSO) in periodontitis and wild‐type mice to test whether inhibiting FAP activity could alleviate periodontitis progression.^[^ ^14^ ^]^ We used Ac‐Gly‐BoroPro as the FAPi, which could selectively inhibit FAP relative to other prolyl peptidases at submicromolar concentrations and with higher biosafety according to previous studies.^[^ ^8^, ^14^, ^32^ ^]^

**Figure 5:**
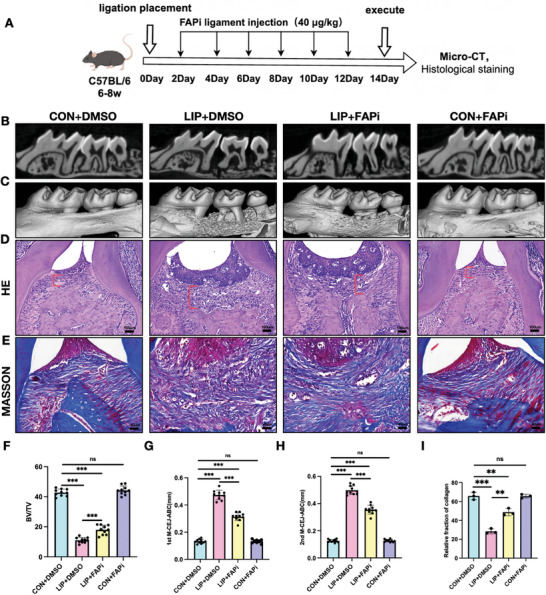
Periodontal injection of FAP inhibitor alleviates periodontal tissue damage in murine periodontitis. A) the strategy establishment of a mouse model of periodontitis treated with FAPi. B,C) Micro‐CT images and 3‐D visualization of the maxilla. D) Representative images of H&E‐stained section. The red line indicates the width of the lamina propria. Scale bar: 100 µm. E) Representative images of the Masson staining section. Scale bar: 40 µm. F–H) Maxilla alveolar bone was quantified by the BV/TV ratio and the cement‐to‐enamel junction to alveolar bone crest (CEJ‐ABC) distance. I) Collagen degradation was quantified by collagen volume fraction. Values are shown as the means ± SD; *n* = 3; ^**^ *p* < 0.01, ^***^ *p* < 0.001, and ^****^ *p* < 0.0001.

After receiving an injection of FAPi or vehicle every other day for two weeks, the mice were sacrificed (**Figure** **5**A). FAP expression significantly decreased in the FAPi‐injected LIP group compared to the vehicle group, indicating the effectiveness of the drug (Figure S5, Supporting Information). We used Cementum enamel junction for alveolar bone crest (CEJ‐ABC) to refer to the degree of alveolar bone resorption and measured the CEJ‐ABC from the alveolar ridge of the second molar to the first molar (1st m) or second molar (2nd m). After injecting FAPi into LIP mice, the alveolar ridge absorption around the second molar teeth was reduced compared with the vehicle group (Figure 5B,F) (Figure S6, Supporting Information). Bone mineral density around the second molar was calculated by the BV/TV ratio, demonstrating a reduction in the overall bone mass loss after FAPi administration (Figure 5C,G,H) (Figure S7, Supporting Information). Further hematoxylin and eosin stains revealed a decreased thickness of the connective gingival tissue after FAPi injection (Figure 5D; red bars spaced apart). Masson staining results showed a decreased extent of gingival collagen fiber degradation (Figure 5E,I). Thus, inhibiting FAP activity alleviates bone loss and collagen degradation in periodontitis.

### 2.6 FAPi Inhibits Macrophage Pro‐Inflammatory Phenotype and Osteoclast‐Oriented Differentiation Phenotype

**Figure 6:**
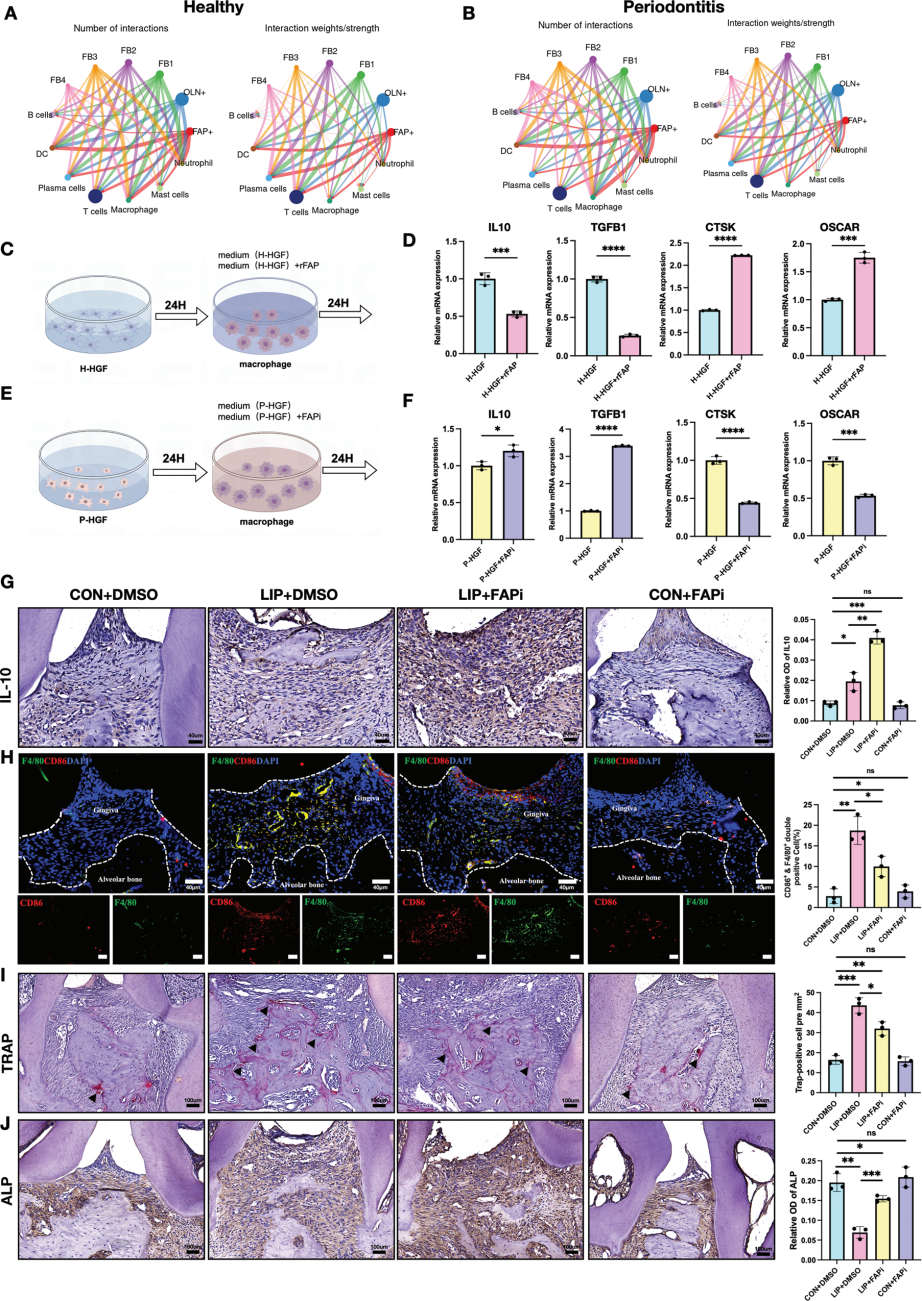
FAPi inhibits macrophage pro‐inflammatory phenotype and osteoclast‐oriented differentiation phenotype. A) The number and strength of signals for different fibroblasts clusters with immune cell clusters in healthy gingiva sample; the color represents the cell clusters, and the width of the wires represents the relative strength and number of interactions. B) The number and strength of signals for different fibroblasts clusters with immune cell clusters in periodontitis gingiva sample. The number of interactions between FAP^+^ fibroblasts with dendritic cells and macrophages exhibited markedly increased in periodontitis condition. C) Experimental design of image D, the supernatant of H‐HGF added with rFAP (100ng mL^−1^) and was used to stimulate macrophages for 24 h followed by qPCR analysis. D) RT‐PCR analysis of anti‐inflammation genes IL10, TGFB1, and osteoclast differentiation genes CTSK and OSCAR in macrophages. E) Experimental design of image F, the supernatant of P‐HGF added with FAPi (5ug mL^−1^) and was used to stimulate macrophages for 24 h followed by qPCR analysis. F) RT‐PCR analysis of anti‐inflammation genes IL10, TGFB1, and osteoclast differentiation genes CTSK and OSCAR in macrophages. G) Representative images of IHC staining and MOD values of IL10, which indicated that FAPi promotes anti‐inflammatory cytokine IL‐10 expression in LIP gingival tissues. Scale bar: 40 µm (H) IF staining of CD86 (red), F4/80 (green), and nuclei (blue) and quantification of double‐positive cells, which suggested that FAPi reduced the number of pro‐inflammatory macrophages in LIP gingival tissues. Scale bar: 40 µm. I) Representative images of TRAP staining and quantification of TRAP‐positive stains were presented as number of TRAP positive areas/mm^2^, decreased number of the osteoclasts in LIP m treated by FAPi compared with LIP group. Black arrows indicated the TRAP‐positive osteoclast cells. Scale bar: 100 µm. J) Representative images of IHC staining and MOD values of alkaline phosphatase (ALP), which demonstrated higher osteogenic activity in LIP treated by FAPi. Scale bar: 100 µm. *n* = 3; ^*^ *p* < 0.05; ^**^ *p* < 0.01; ^***^ *p* < 0.001; and ^****^ *p* < 0.0001. MOD: mean optical density.

FAP influences bone homeostasis by directly degrading collagen fibers in bone tissue or indirectly regulating the differentiation and formation of osteoclasts.^[^ ^8^ ^]^ We analyzed the number and strength of signaling pathways between FAP^+^ fibroblast subpopulations and immune cells under healthy and periodontitis conditions to investigate how FAP^+^ fibroblasts communicate with other immune cells. Interestingly, the communication strength between FAP^+^ fibroblasts and all immune cell types remained comparable under both conditions. However, regarding communication frequency, FAP^+^ fibroblasts exhibited markedly increased interactions with dendritic cells and macrophages, which was more pronounced in periodontitis (**Figure** **6**A,B). Since the pro‐inflammatory activation of macrophages and osteoclast differentiation are critical in periodontitis progression,^[^ ^33^ ^]^ FAP may play a role in macrophage activation during periodontitis. Then, we performed in vitro experiments using human recombinant FAP protein (rFAP) and FAPi to further explore the role of FAP in the regulation of macrophage function (Figure 6C,D). Specifically, an H‐HGF‐conditioned medium was filtered and supplemented with rFAP to stimulate macrophages, while a P‐HGF‐conditioned medium was filtered and supplemented with FAPi. Under stimulation with the rFAP‐supplemented H‐HGF medium, macrophages exhibited significantly elevated mRNA expression of osteoclast differentiation markers, such as CTSK and OSCAR, while the expression of repair‐associated genes, including IL10 and TGFB1, was significantly reduced (Figure 6E). Conversely, under stimulation with the FAPi‐supplemented P‐HGF medium, macrophages showed increased IL10 and TGFB1 mRNA expression, while CTSK and OSCAR mRNA levels were notably decreased compared to the stimulation with the P‐HGF medium alone (Figure 6F). Therefore, FAPi effectively inhibits FAP activity, reducing the pro‐inflammatory and osteoclast‐promoting phenotypes of macrophages. Furthermore, in vivo experiments using FAPi‐treated LIP mouse models showed significantly increased IL‐10 expression in gingival tissues following a FAPi injection (Figure 6G). Additionally, the number of pro‐inflammatory CD86^+^ macrophages was markedly reduced (Figure 6H). Afterward, we quantified the number of osteoclasts and alkaline phosphatase (ALP) activity to assess the effects of FAPi on the osteogenic microenvironment. FAPi treatment significantly reduced osteoclast numbers (Figure 6I) and increased ALP activity, indicating enhanced osteogenesis (Figure 6J). Hence, FAPi improves the transition from a pro‐osteoclastic to a pro‐osteogenic microenvironment in periodontitis. In summary, FAP contributes to periodontitis progression by promoting the pro‐inflammatory phenotype of macrophages and enhancing osteoclast differentiation, while FAP inhibition offers a promising strategy to mitigate alveolar bone resorption and inflammation in periodontitis.

## 3 Discussion

**Figure 7:**
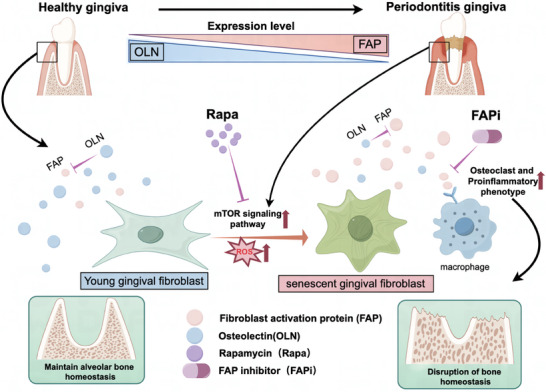
Schematic diagram of the mechanism by which the imbalance of FAP and OLN expression in fibroblasts promotes the progression of periodontitis. The elevated ROS in periodontitis causes excessive activation of the mTOR signaling pathway in fibroblasts, accelerating cellular senescence. Senescent fibroblasts are featured by overexpression of FAP and decreased expression of OLN, which promote macrophages to obtain pro‐inflammatory and osteoclast phenotypes, thereby exacerbating bone resorption in periodontitis.

Periodontitis is a chronic inflammatory disease that destroys soft and hard tissues. Although mechanical debridement remains a routine treatment for periodontitis, adjunctive therapies targeting excessive host immune responses represent effective strategies. However, the clinical application of nonsteroidal anti‐inflammatory drugs is often limited by the risk of severe adverse reactions.^[^ ^34^ ^]^ Serine proteases contribute to tissue destruction associated with periodontitis,^[^ ^9^ ^]^ yet the specific role of FAP, as a serine protease, in periodontitis has not been explored. This study identified a positive genetic causal relationship between increased FAP expression and the risk of periodontitis. Additionally, we observed a disruption in the balance of FAP and OLN expression in periodontitis tissues. Elevated FAP expression drove macrophage polarization toward a pro‐inflammatory and bone‐resorbing phenotype. Mechanistically, gingival fibroblast senescence disrupted the FAP/OLN balance by activating the mTOR pathway. Furthermore, we demonstrated that a selective small‐molecule FAPi effectively alleviated periodontal tissue damage. Overall, senescent gingival fibroblasts disrupt the FAP/OLN balance via the mTOR signaling pathway, playing a critical role in periodontitis progression. Thus, FAP inhibition may represent a promising therapeutic strategy for periodontitis treatment (**Figure** **7**).

We found that disrupting the FAP and OLN balance contributes to the destruction of soft and hard tissues in periodontitis. A previous study demonstrated that FAP and OLN could interact and function together.^[^ ^14^ ^]^ In that study, we preliminarily predicted the 3D structure of the FAP/OLN complex to understand their functional regulation and develop small‐molecule drugs targeting FAP. However, further experimental validation is required to determine their precise chemical structure and binding mechanism. Previous studies demonstrated that FAP could degrade degenerative Col I and native Col I precleaved by MMP1.^[^ ^35^, ^36^ ^]^ Consistent with these findings, we observed that pharmacological inhibition of FAP in periodontitis mouse models decreased collagen fiber degeneration. FAP and OLN also regulate the osteogenic differentiation of bone mesenchymal stem cells (BMSCs). Specifically, OLN promotes bone formation by facilitating BMSC differentiation into mature osteoblasts,^[^ ^37^ ^]^ while FAP inhibits bone formation by suppressing the Wnt/β‐catenin signaling pathway in BMSCs.^[^ ^8^ ^]^ Additionally, FAP can impair BMSC osteogenic differentiation by activating the expression of histone lysine demethylase 7A.^[^ ^38^ ^]^ Notably, we observed that FAP^+^ fibroblasts and OLN^+^ fibroblasts were predominantly localized near the alveolar bone surface, where bone resorption typically begins in periodontitis. Thus, OLN^+^ fibroblasts, which are abundant in healthy tissues, might form a protective layer on the alveolar bone by inhibiting FAP activity. However, the significant increase in FAP^+^ fibroblasts is likely to delay bone repair in periodontitis. This critical shift drives the destruction of alveolar bone during periodontitis progression. Taken together, different fibroblast subpopulations are involved in maintaining the dynamic balance between FAP and OLN in periodontal tissues, providing new insights into the role of fibroblasts in the regulation of periodontal homeostasis.

FAP also plays a role in regulating macrophage phenotype, thereby influencing the immune environment. On one hand, previous studies demonstrated that FAP induces macrophages to secrete pro‐inflammatory cytokines, such as IL‐6 and TNF‐α,^[^ ^39^, ^40^ ^]^ accompanied by the significant activation of the NF‐κB pathway.^[^ ^8^ ^]^ NF‐κB activation is a critical step driving the polarization of macrophages toward a pro‐inflammatory phenotype.^[^ ^41^ ^]^ On the other hand, FAP promotes the differentiation of macrophages into TRAP^+^ osteoclasts in a dose‐dependent manner via the NF‐κB‐RANKL/RANK axis.^[^ ^8^ ^]^ Consistent with these findings, we observed that FAPi, administered in vitro and in vivo, decreased the proportion of pro‐inflammatory macrophages and TRAP^+^ osteoclasts. Notably, FAPi treatment also enhanced the expression of macrophage repair–related genes, such as IL‐10 and TGF‐β, fostering a pro‐regenerative environment in periodontitis. Investigating the specific mechanisms by which FAP regulates macrophages in periodontitis is highly important and could provide valuable insights for therapeutic development.

FAP could serve as a potential marker of senescent cells. During cellular senescence, sustained growth arrest and changes in protein expression are accompanied by the SASP.^[^ ^42^ ^]^ In osteoarthritis, single‐cell sequencing identified key pathogenic chondrocyte subpopulations characterized by high FAP expression alongside SASP‐related genes.^[^ ^20^ ^]^ Similarly, our experiments demonstrated that FAP expression paralleled that of senescence markers, such as P21, in the gingival cell senescence model. Furthermore, the imbalance of FAP and OLN expression observed in periodontitis gingival tissues was mitigated following in vivo clearance of senescent cells using ABT263. Single‐cell analysis further revealed that FAP^+^ fibroblasts exhibit not only activation of cellular senescence pathways but also significant activation of the NF‐κB pathway, a central regulator of SASP.^[^ ^43^, ^44^ ^]^ Therefore, targeting senescent gingival fibroblasts via FAP may represent a promising therapeutic strategy. However, while many studies explored the role of FAP in cellular senescence, the mechanisms by which senescence drives FAP expression remain unclear. This study showed that cellular senescence induced by the inhibition of DNA replication and elevated ROS levels leads to FAP overexpression, establishing cellular senescence as a significant driver of FAP expression. Moreover, we observed significant enrichment of the mTORC1 pathway during the transition from OLN^+^ fibroblasts to FAP^+^ fibroblasts. Inhibiting the mTOR pathway using rapamycin not only suppressed cellular senescence but also restored the balance between FAP and OLN expression in senescent gingival fibroblasts. Hence, gingival fibroblast senescence drives FAP expression via the mTOR pathway. Consequently, targeting the mTOR pathway could be a pivotal strategy for restoring the balance between FAP and OLN expression in fibroblasts.

Intriguingly, periodontitis is characterized by a significantly increased uptake of [68Ga] Ga‐FAPI‐04 compared to normal physiological uptake, with uptake levels surpassing those of 18F‐FDG (mean SUV_max_: FAPI: 3.7 ± 0.9 versus FDG: 2.8 ± 0.3, *n* = 33).^[^ ^17^ ^]^ These findings provide a strong theoretical basis for designing FAP‐targeting diagnostic molecules and therapeutic agents for periodontitis. For instance, a recent study has demonstrated the successful application of lipid nanoparticles loaded with FAP‐specific small interfering RNA (LNPs@FAP siRNA), which mitigated osteoarthritis progression through their sustained‐release properties.^[^ ^20^ ^]^ This strategy offers promising avenues for developing slow‐release drugs and delivery platforms tailored for periodontal pockets. In this study, we utilized Ac‐Gly‐BoroPro, a highly selective FAP inhibitor with a selectivity ninefold greater for FAP than for its homolog DppIV.^[^ ^45^ ^]^ Systemically administering Ac‐Gly‐BoroPro in mice significantly increased the trabecular bone volume and density at the metaphysis of the femur in a dose‐dependent manner without observable side effects.^[^ ^8^ ^]^ Furthermore, we successfully alleviated inflammation and alveolar bone resorption in the mouse model of periodontitis through localized injections of Ac‐Gly‐BoroPro into the periodontal tissues. However, further studies are required to determine the optimal concentration and delivery route for FAP inhibitors in the treatment of periodontitis, along with large animal and clinical trials to validate their safety and therapeutic efficacy. In conclusion, our findings unveil a novel molecular mechanism by which senescent gingival fibroblasts regulate the balance between FAP and OLN through the mTOR signaling pathway. This study highlights FAP as a critical immune regulator in the pathogenesis of periodontitis and underscores its potential as a promising therapeutic target for periodontitis.

## 4 Experimental Section

### Mendelian Randomization

The Prolyl endopeptidase FAP protein data used in this study come from a published gene wide association studies (GWAS) data that included 3301 participants of European ancestry.^[^ ^22^ ^]^ The data of periodontitis from The Gene‐Lifestyle Interactions in Dental Endpoints collaboration provided summary statistics for periodontitis with a total of 17353 clinical cases and 28210 controls, based on 7 European descent cohort studies.^[^ ^46^ ^]^ First, single nucleotide polymorphisms (SNPs) associated with FAP protein that met the significance threshold of *p* < 1 × 10^−6^ were selected as potential IVs, and a clumping procedure was performed to set the linkage disequilibrium (LD) coefficient *R* ^2^ to less than 0.001 in a 10 000 kb window, to determine the independent SNPs. Traditional Mendelian randomization (MR)analysis was then performed using the inverse variance weighting (IVW) method which was primarily utilized because of its high statistical efficiency and common application.^[^ ^47^ ^]^ Furthermore, IVW method was used to determine the presence of heterogeneity using the Cochran Q test, with *p* > 0.05 indicating the absence of heterogeneity. MR‐Egger regression was used to assess the potential horizontal pleiotropy of SNPs as IVs.^[^ ^48^ ^]^ An overall *p* < 0.05 MR‐Egger analysis indicated the presence of horizontal pleiotropy. Finally, a leave‐one‐out sensitivity analysis was performed, and MR results were considered stable if the removal of any IVs did not result in a significant change in the results.^[^ ^49^ ^]^ All results are shown as OR and 95% confidence intervals (CI), with differences considered statistically significant at *p* < 0.05. All analyses in this MR study were performed using the R software packages “TwoSampleMR”.

### Structural Bioinformatics

The AlphaFold3 server was used to predict the interaction patterns between FAP (NP_004451.2) and OLN (NP_002966.1).^[^ ^50^ ^]^ Pymol 2.2.0 software was used for visualization and hydrogen bond between FAP and OLN was indicated by the red line.

### Human Samples

A total of 12 subjects (healthy group: *n* = 6, aged 22–33 years old; periodontitis group: *n* = 6, aged 23–46 years) were included in this study. All subjects provided written informed consent. This study has been approved by the Ethics Committee of School & Hospital China Hospital of Stomatology Wuhan University (No. WDKQ2024B01). Healthy control group gingival tissue was obtained from patients who underwent crown lengthening or tooth extraction. The inclusion criteria were as follows: 1) age 18–50 years old; 2) good general health without systemic diseases; 3) gingival tissue without erythema, edema, bleeding, and other symptoms; 4) no use of nicotine‐related products in the past 6 months. Gingival tissue from periodontitis group included patients who underwent periodontal pocket resection. Inclusion criteria for patients with chronic periodontitis were as follows: 1) age 18–50 years old; 2) good general health, no systemic disease, and tolerance to periodontal surgery; 3) patients diagnosed with periodontitis (stage III/IV periodontitis);^[^ ^51^ ^]^ 4) no use of nicotine‐related products within the past 6 months. Healthy and periodontitis gingival tissue samples (*n* = 3) were collected for further histological staining analysis, and primary gingival fibroblasts were extracted and cultured from healthy and periodontitis gingival tissue samples (*n* = 3).

### Animal Operations and Grouping

The ligature model was effectively used to induce alveolar bone resorption in C57BL/6 mice. C57BL/6 mice have been widely used in constructing periodontitis models, particularly in the ligature‐induced periodontitis (LIP) model. The LIP model is one of the most representative models of periodontitis in humans for short‐term experiments.^[^ ^52^ ^]^ C57BL/6 mice (8 weeks, male) were purchased and bred. Animal testing in this study followed the guidelines established by the Animal Research Ethics Committee of Wuhan University School of Stomatology and Hospital, China (No. S07924040B) and followed ARRIVE Guideline 2.0. Animals were housed in an environment free of specific pathogens, temperature/humidity controlled, and a light/dark cycle of 12 h. After anesthesia, the ligation‐induced periodontitis (LIP) group was ligated with 5‐0 silk thread (SA82G; ETHICON) around the second molars on both sides of the upper jaw.^[^ ^53^ ^]^ Molar ligation induces periodontitis and bone loss due to significant local bacterial accumulation. The ligatures were inspected daily to ensure they remained in place throughout the experiment. After 14 days of ligation, total protein and RNA were extracted from the gingival tissues of the bilateral maxilla in both the ligation group and the control group (*n* = 3 mice). Histological analysis was performed on unilateral maxilla samples from another set of mice (*n* = 3).

To investigate the role of cellular senescence in the expression of FAP and OLN during periodontitis, mice with ligature‐induced periodontitis (LIP) received intraperitoneal injections of either the vehicle (10% DMSO, 40% PEG300, 5% Tween‐80, and 45% saline) or the senolytic drug ABT263 (50 µg kg^−1^ day^−1^; HY‐10087, MCE). The mice were divided into two groups: LIP + VEHICLE and LIP + ABT263. Three days after ligation, the LIP + ABT263 group received ABT263 for two 4‐day cycles with a 3‐day break in between, while the VEHICLE group received an equivalent volume of saline. After 14 days of ligation, the mice were euthanized, and their maxillae and gingiva were collected for histological staining.

The therapeutic effect of a FAP inhibitor (FAPi) on periodontitis was evaluated, and mice were randomly divided into four groups (*n* = 5): CON + DMSO, LIP + DMSO, LIP + FAPi, and CON + FAPi. In the LIP + FAPi and CON + FAPi groups, 20 µL of FAPi (40 µg kg^−1^; HY‐101801, MCE) was injected into the gingival tissues every two days, while the other groups received an equivalent volume of DMSO as a control. After 14 days of ligation, the mice were euthanized. Their maxillae and gingival tissues were collected for micro‐CT scanning (10 unilateral maxillae) and histopathological analysis (3 unilateral maxillae).

### Micro‐Computed Tomographic (Micro‐CT) Scanning and Analysis

Micro‐CT scans were performed using Bruker Micro‐CT SkyScan 1276 (Konitich, Germany). Region of interest (ROI) was established in three dimensions: vertical direction, from 0.2 mm to the cement‐enamel junction (CEJ) of the second molar, extending 0.5 mm to the apex; mesial‐distal direction, from the nearest middle surface of the cement‐enamel junction of the first molar (1st m) to the root bifurcation of the third molar (3rd m); buccolingual and lingual sides, within 1.5 mm around the root bifurcation of the second molar. The ratio of bone volume to total volume (BV/TV) was calculated based on this ROI. CEJ vertical distances from the anterior and posterior alveolar crest (CEJ‐ABC) of the second molar to the first molar (lst) and the second molar (2nd) were measured. 3D reconstruction, calculations, and measurements were finally performed using object research systems (ORS) Dragonfly software (version 2022.1, Montreal, Canada).

### Histopathological and Immunohistochemical (IHC) Examinations

Human gingival samples and mice maxillary samples were fixed in 4% neutral formalin for 24 h and then decalcified with 15% EDTA at pH 7.4 for 4 weeks. The decalcification solution was refreshed every 2 days. The tissue was then trimmed, paraffin‐fixed, and dehydrated. Hematoxylin and eosin (H&E) staining, Masson's (G1120; Solarbio), and TRAP (G1050‐ 50T; Servicebio) kits were performed according to the manufacturer's instructions. For IHC staining, protein expression was determined by staining with the following primary antibodies: FAP (M079003; Abmart), OLN (IPB5993; Baijia), IL‐10 (60269‐1‐Ig; Proteintech) and ALP (11187‐1‐AP; Proteintech). Tissue sections were observed with the diaminobenzidine substrate kit (ZLI‐9018; Zhongshan Jinqiao Biotechnology) and nuclear staining was performed with hematoxylin. Images were captured using a microscope (Olympus Corporation, Japan). The interdental gums region was selected as the study area. For semi‐quantification of protein expression, the mean optical density (MOD) of the positively stained areas was measured using imageJ2 software (version: 2.14.0, National Institutes of Health). Calculation of TRAP‐positive cell numbers and collagen volume fractions (which were stained into blue) for individual sections was also measured by imageJ2 software.

### Immunofluorescent Staining

For immunofluorescent analysis, HGF was seeded on confocal cell culture dishes for immunofluorescence staining. First, harvested samples were cell‐fixed for 10 min using 4% paraformaldehyde. Second, cells were placed in 0.1%Triton X‐100 for cell permeabilization for 10 min and 5% BSA for blocking for 1 h at room temperature. Paraffin sections were blocked with 5% BSA after antigen retrieval. Primary antibodies used for immunofluorescence included FAP, OLN, P16 (10883‐1‐Ap; Proteintech), VIM (A19607; ABclonal), CD86(APC‐65165; Proteintech), and F4/80 (Ab16911; Abcam). Anti‐mouse cy3 red (AS008; ABclonal) and anti‐rabbit 488 nm green secondary antibodies (AS073; ABclonal) were to label primary antigens. Nuclei were stained with 4‐6‐diamidino‐2‐phenylindole (DAPI) (Zhongshan Biotechnology, Ltd, China). The stained sections were examined using an Olympus DP72 microscope (Olympus, Japan).

### RT‐qPCR Analysis

Total RNA was collected using KarrolTM RNA Reagent (K3102; Karroten) and total RNA concentration was measured using a Nanodrop2000 instrument (Thermo Fisher Scientific, Waltham, MA, United States). Total RNA was reverse transcribed into cDNA using qPCR RT Master Mix (RK20429; ABclonal) according to the manufacturer's guidelines. Universal SYBR Green Fast qPCR Mix (RK21203; ABclonal) and LightCycler 480 Real‐Time PCR system (Roche; Switzerland) were used for real‐time PCR chips. Gene expression was calculated by CT method and normalized by β‐actin. The relative number of target genes was evaluated by 2^−ΔΔCt^ method and calibrated with control group. Primer sequences are listed in Table S1 (Supporting Information).

### Western Blot Analysis

Proteins were extracted from human samples or primary gingival fibroblasts and dissolved in 80 µL RIPA buffer to extract total protein, supplement protease, and 1% phosphatase inhibitor. BCA protein detection kit (P0012; Beyotime Biotechnology) was used. Proteins were separated by sodium dodecyl sulfate‐polyacrylamide gel electrophoresis (SDS‐PAGE) and transferred to polyvinylidene fluoride (PVDF) membranes (ISEQ00010; Millipore). After blocking the membrane with a primary antibody blocking solution, the primary antibodies in this study include anti‐β‐ACTIN (AC004; ABclonal), anti‐FAP, anti‐OLN, anti‐P16, anti‐P21 (10355‐1‐AP; Proteintech), anti‐P‐MOTR (T56571F; Abmart), and anti‐MOTR (IPB0534; Baijia). Secondary antibodies include: HRP Goat Anti‐Rabbit IgG (H+L) (AS014; ABclonal) and HRP Goat Anti‐Mouse IgG (H+L) (AS003; ABclonal). Protein bands were visualized using the Western Bright ECL HRP substrate Kit (Advansta, USA). The relative protein expression was normalized to β‐ACTIN.

### Single‐Cell RNA Sequencing Analysis

Single‐cell RNA sequencing datasets including GSE171213 and GSE164241 were obtained from the GEO dataset.^[^ ^23^, ^24^ ^]^ GSE171213 dataset contains samples from 4 healthy individuals, 5 periodontitis individuals, and 3 periodontitis after treatment individuals. GSE164241 dataset contains samples from 13 healthy individuals and 8 periodontitis individuals. Standard procedures for single cell data processing were performed according to Seurat's official website. Briefly, a single‐cell analysis object is created using the CreateSeuratObject function, filtered by Feature_RNA > 200 & MT_percent < 40 for GSE171213 and Feature_RNA > 200 & nFeature_RNA < 5000 & MT_percent < 10 & nCount_RNA < 25000 & nCount_RNA > 1000 for GSE164241. 47892 and 74725 cells were obtained by removing batch processing effects between samples and Single‐cell data were integrated using “SplitObject”, “lapply”, “NormalizeData”, “FindVariableFeatures”, “SelectIntegrationFeatures”, “findinintegrationanchors”, “IntegrateData” and other functions. Then “FindNeighbors”, “FindClusters” and “RunUMAP” were applied to reduce dimensionality in single‐cell objects for cell aggregation. “FindAllMarkers” function was used to label genes for each cell cluster. Gene ontology (GO), Kyoto Encyclopedia of Genes and Genomes (KEGG) and Gene set enrichment analysis (GSEA) were performed using R package “ClusterProfiler(Version:4.6.2)”.^[^ ^54^ ^]^ Cell‐cell communication analyses were conducted using R package “Cellchat(Version:1.6.1)”. The single‐cell RNA‐seq results were visualized using “DimPlot”, “DotPlot”, “VlnPlot”, “DittoBarPlot”, “dittoDimHex”, “dittoDotPlot”, “ggscatterstats” functions.

### Primary Gingival Fibroblast Cell Isolation and Culturing

Gingival tissue was transported from the clinic to the laboratory with phosphate buffered saline (PBS), and human gingival tissue was rinsed repeatedly in large quantities (3–4 times) with pre‐cooled PBS, and pulverized to a size of 1 mm^2^.^[^ ^55^ ^]^ The tissue pieces were placed in cell culture dishes and the cell fragments were immersed in Type II Collagenase (2275GR001; BioFroxx) for 2 h 37 °C and incubated for 5–7 days in DMEM high glucose medium (YC‐2067; Gibco) containing 20% fetal bovine serum (ST40‐37500; PAN‐SERATECH) at 37 °C and 5% CO_2_. When the cells reached 80%–100% fusion, they were passaged with 0.25% trypsin(25200056; Gibco), and the GFs of passages 3–10 were used for subsequent experiments. To simulate the periodontitis environment in vitro cells were also stimulated with pg‐LPS (1ug/mL) (InvivoGen, San Diego, CA) At different times of stimulation. Subsequently, HGFs cells were harvested for subsequent RNA.

### Cell Drug Administration

HGF (150000 cells per milliliter using a hematocytometer) was inoculated into a 3 mL culture dish and incubated overnight in a complete culture medium at 37 °C. For bleomycin (BLM), different concentrations (0–100 µm) of bleomycin (S1214, Selleck) were used to stimulate HGF 12 h to induce DNA‐loss cell senescence models. For hydrogen peroxide(H_2_O_2_), HGF was treated with H_2_O_2_ at different concentrations (0–400 µm) and different times (0–24 h) to induce cell senescence. Using the Cell Counting Kit‐8 (CCK‐8) assay, the inhibition rate of cell proliferation was measured, and the optimal condition was identified, where the proliferation inhibition rate was 50–70%, this corresponded to a concentration of 100 µm and a stimulation duration of 12 h for H_2_O_2_, and 50 µm for bleomycin. After removing hydrogen peroxide stimulation, HGF cells were cultured  for another 12 h. Alternatively, different concentrations (0–400 nm) of rapamycin (ABS47000712; Absin) were added to treat HGF for 4 h before collecting HGF cells for subsequent experiments. An optimal condition of 100 nM for 4 hours was established through statistical analysis (ANOVA test), which compared cell proliferation rates across experimental groups and the control group.

Human monocyte THP‐1 (TIB‐202; ATCC) was cultured in RPMI 1640 (11875119; Gibco) supplemented with 10% FBS and 1% penicillin G/ streptomycin. THP‐1 cells were induced into macrophages by 200ng mL^−1^ PMA (P1585; Sigma) for 48 h.^[^ ^56^ ^]^ To mimic the effect of FAP from HGF THP‐1 cells were stimulated with supernatant of healthy HGF culture medium or addition with recombined FAP protein (rFAP, 100ng mL^−1^, UA010165; STARTER). Furthermore, supernatants from periodontitis HGF cell cultures, with or without the addition of FAPi (5 µg mL^−1^), 17 were employed to treat THP1 cells to elucidate the role of FAP originating from periodontitis‐HGF. After 24 h stimulation, THP‐1 cells were then harvested for qPCR analysis.

### Cell Proliferation Assay

Cell Count Kit‐8 (CCK‐8) (BS350A, Biosharp) was used to measure cell proliferation. Briefly, 5 × 10^3^ of healthy HGF cells were seeded in 96‐well plates and cultured for 24 h. After stimulation with H_2_O_2_ or rapamycin at different concentrations and times, CCK‐8 reagent was then added to the plate before and after restimulation and incubated at 37 °C for an additional 1.5 h. Finally, the 96‐well plate was read at a wavelength of 450nm. OD values were compared, and cell viability expressed as a percentage (%) of control.

### Staining for Senescence‐Associated Galactosidase (SA‐β‐gal)

SA‐β‐gal staining was performed using the senescent β‐galactosidase staining (C0602; Beyotime Biotechnology) and incubated overnight at 37 °C for 12 h in a CO_2_‐free chamber according to manufacturer's instructions. Positive, blue‐stained cells were counted under a common light microscope (DP72 microscope, Olympus, Japan). Results are expressed as a percentage of positive cells. Six regions of interest were randomly captured from each group and positive cells were counted by Image J v2.0 (NIH, Bethesda, MD, USA).

### Detection of Mitochondrial Reactive Oxygen Species (ROS)

Cytoplasmic ROS were detected with DCFH‐DA fluorescent probe (S033S; Beyotime Biotechnology). Cells were exposed to 10 µM DCFH‐DA fluorescent probe (488 nm excitation, 525 nm emission) in DMEM medium for 20 min at 37 °C and samples were protected from light. Nuclei were incubated with Hoechst staining solution (C1011; Beyotime Biotechnology) for 20 min at 37 °C in the dark. Cell fluorescence was detected using an inverted phase contrast microscope (Olympus IX83; Japan) and followed by image processing using Image J v2.0 (NIH, Bethesda, MD, USA).

### Statistical Analysis

“SPSS 19.0”, “Image J” and “GraphPad Prism 6.0” software was used for data analysis, image processing, and statistical mapping, respectively. Data are expressed as mean ± standard deviation. The *t*‐test or analysis of variance (ANOVA) was used for group comparisons that obeyed normal distribution, and the nonparametric rank sum test was used for group comparisons that did not obey normal distribution. The experiment was repeated at least 3 times, *p* < 0.05 was statistically significant. ^*^ *p* < 0.05, ^**^ *p* < 0.01, ^***^ *p* < 0.001, and ^****^ *p* < 0.0001.

## Conflict of Interest

The authors declare no conflict of interest.

## Supporting information



Supporting Information

## Data Availability

The data that support the findings of this study are available from the corresponding author upon reasonable request.
