# Supplementary material for: Senescent Fibroblasts Drive FAP/OLN Imbalance Through mTOR Signaling to Exacerbate Inflammation and Bone Resorption in Periodontitis
Source: Adv Sci (Weinh). 2024 Dec 24;12(7):2409398. doi: 10.1002/advs.202409398 (PMC11831441; doi:10.1002/advs.202409398)
Supplement: Supplementary file 1 — Supporting Information [file ADVS-12-2409398-s001.pdf]

ADVANCED SCIENCE
Open Access

Supporting Information

for *Adv. Sci.*, DOI 10.1002/advs.202409398

Senescent Fibroblasts Drive FAP/OLN Imbalance Through mTOR Signaling to Exacerbate Inflammation and Bone Resorption in Periodontitis

Chenghu Yin, Liangliang Fu, Shuling Guo, Youde Liang, Taizhi Shu, Wenjun Shao, Haibin Xia, Ting Xia and Min Wang**

Senescent fibroblasts drive FAP/OLN imbalance through mTOR signaling to exacerbate inflammation and bone resorption in periodontitis

Chenghu Yin, Liangliang Fu, Shuling Guo, Youde Liang, Taizhi Shu, Wenjun Shao, Haibin Xia, Ting Xia, Min Wang**

Supporting Information

Table S1. Primer sequences for real-time PCR.. 2
Table S2. Results of horizontal pleiotropy by the MR-Egger intercept test............... 2
Table S3. Results of heterogeneity by the Cochran's Q test....................................... 2
Figure S1. A leave-one-out approach was used to assess the over-representation of individual SNPs of FAP on the Mendelian randomization analysis.. 3
Figure S2. Predicted interaction patterns by analyzing the binding regions and functional domains of FAP and OLN by AlphaFold3... 3
Figure S3. The imbalanced expression of FAP/OLN in gingival fibroblasts was found in the periodontitis.. 4
Figure S4. Cell viability, SA-β-gal Staining, RT-PCR and Western blot analysis of H-HGF with or without bleomycin stimulation.. 5
Figure S5. Representative images of IHC staining and MOD values of FAP................................ 5
Figure S6. All the Micro-CT images from the experimental mice in Figure 5B............................ 6
Figure S7. All the 3-Dimensional visualization of the maxilla images from the experimental mice in Figure 5C.. 7

Table S1. Primer sequences for real-time PCR

Gene	5'-3'	Primer Sequences (5'- 3')
Mouse-Fap	Forward Primer	GTCACCTGATCGGCAATTTGT
	Reverse Primer	CCCCATTCTGAAGGTCGTAGAT
Mouse-Oln	Forward Primer	TTCTCCCACACCAGAGGACACT
	Reverse Primer	CAACCACACGGGTGTCCAAAAC
Mouse-β-actin	Forward Primer	AGATGACCCAGATCATGTTTGAGA
	Reverse Primer	AGAGCCACCAATCCACACAG
Human-FAP	Forward Primer	TGGCGATGAACAATATCCTAGA
	Reverse Primer	ATCCGAACAACGGGATTCTT
Human-OLN	Forward Primer	ACACCCGCGATGCCGTGCAAG
	Reverse Primer	CGAGAGCAGGAAGCACTTGTGG
Human-P16^{CDKN2A}	Forward Primer	CTCCGGAAGCTGTCGACTTC
	Reverse Primer	TTCTGCCATTTGCTAGCAGTGT
Human-P21^{CDKN1A}	Forward Primer	CGATGGAACTTCGACTTTGTCA
	Reverse Primer	GCACAAGGGTACAAGACAGTG
Human-IL10	Forward Primer	TCTCCGAGATGCCTTCAGCAGA
	Reverse Primer	TCAGACAAGGCTTGGCAACCCA
Human-TGFB	Forward Primer	TACCTGAACCCGTGTTGCTCTC
	Reverse Primer	GTTGCTGAGGTATCGCCAGGAA
Human-CTSK	Forward Primer	GAGGCTTCTCTTGGTGTCCATAC
	Reverse Primer	TTACTGCGGGAATGAGACAGGG
Human-OSCAR	Forward Primer	GCAGCGAGGTGCTGGTCATCA
	Reverse Primer	ACTGCGCCAGTCAAAAGTGACC
Human-β-ACTIN	Forward Primer	CAGGGCGTGATGGTGGGCA
	Reverse Primer	CAAACATGATCTGGGTCATCTTCTC

Table S2. Results of horizontal pleiotropy by the MR-Egger intercept test

**Exposure**	**Outcome**	**Intercept**	**SE**	***P*-value**
Prolyl endopeptidase FAP	periodontitis	0.009	0.027	0.766

Table S3. Results of heterogeneity by the Cochran's Q test

**Exposure**	**Outcome**	**Method**	**Q**	**Q_df**	**Q_pval**
Prolyl endopeptidase FAP	periodontitis	MR Egger	2.529	3	0.470
		Inverse variance weighted	2.634	4	0.621

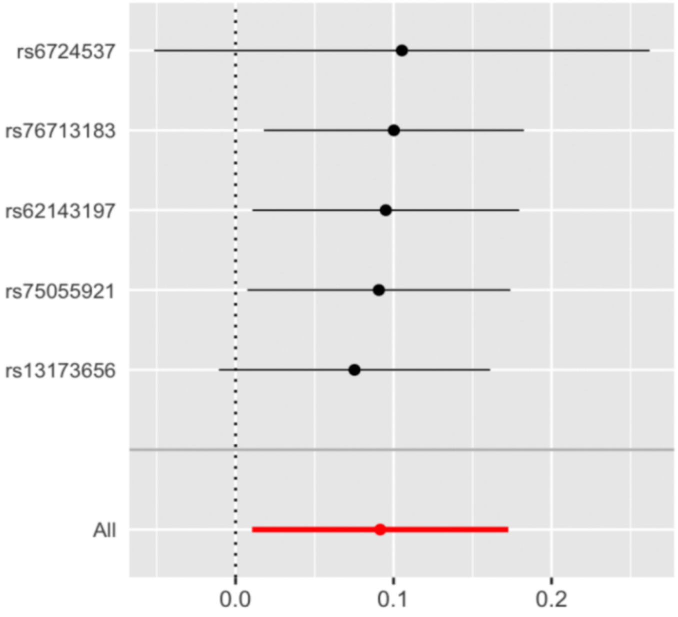

Figure S1. A leave-one-out approach was used to assess the over-representation of individual SNPs of FAP on the Mendelian randomization analysis, which demonstrated the stability of the MR findings.

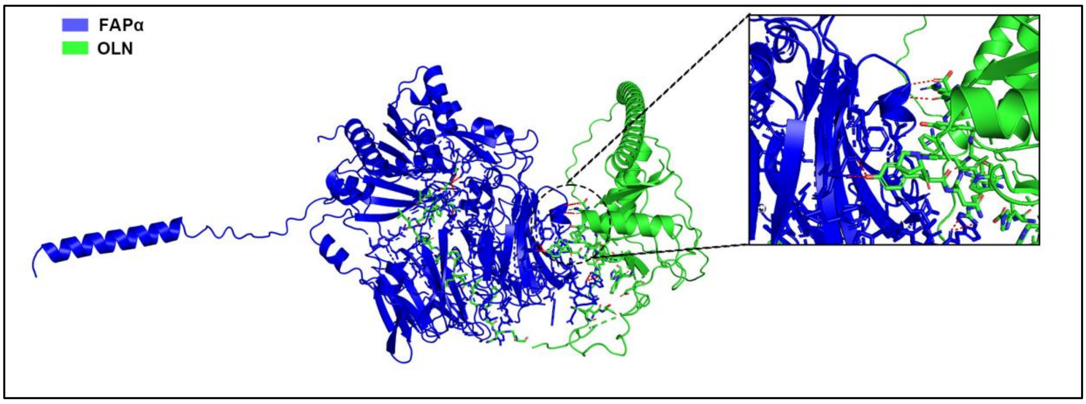

Figure S2: Predicted interaction patterns by analyzing the binding regions and functional domains of FAP and OLN by AlphaFold3.

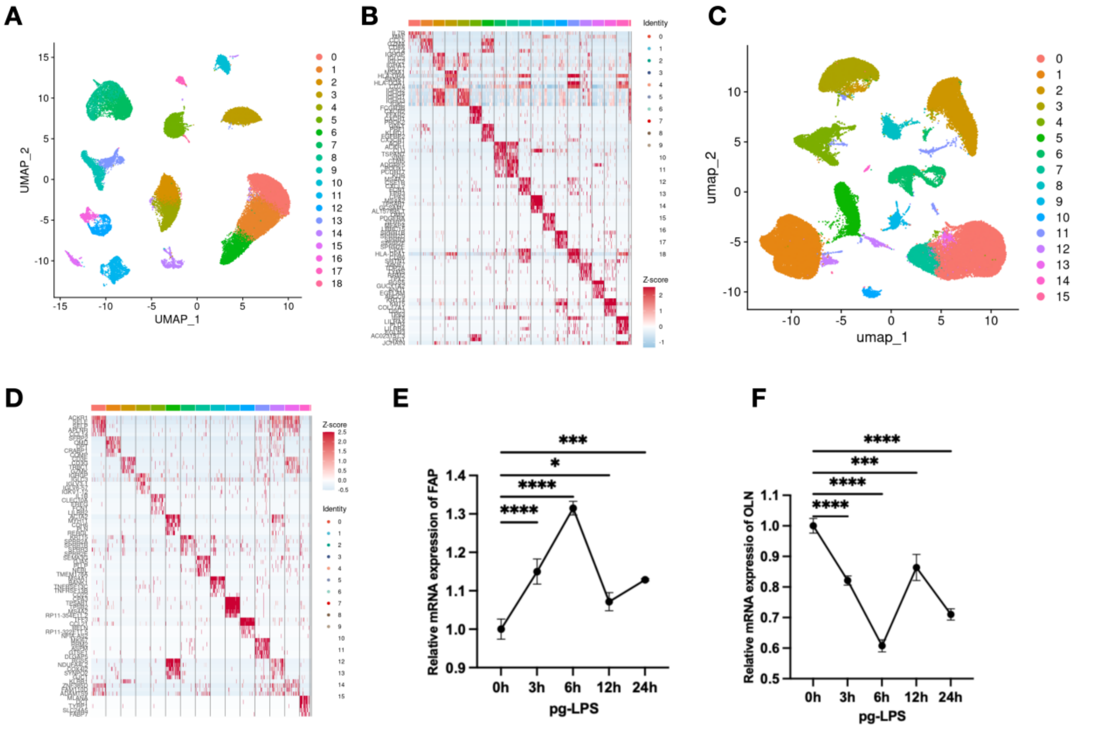

Figure S3. (A) UMAP diagram and single-cell annotation of cells clusters for the healthy and periodontitis samples from GSE171213. (B) The heatmap showing markers for each cluster in GSE171213. (C) UMAP diagram and single-cell annotation of cells clusters for the healthy and periodontitis samples from GSE164241. (D) The heatmap showing markers for each cluster in GSE164241. (E, F) qPCR analysis of FAP and OLN expression in pg-LPS stimulated primary human gingival fibroblasts. $n = 4$; *$P < 0.05$, **$P < 0.01$, ***$P < 0.001$, ****$P < 0.0001$.

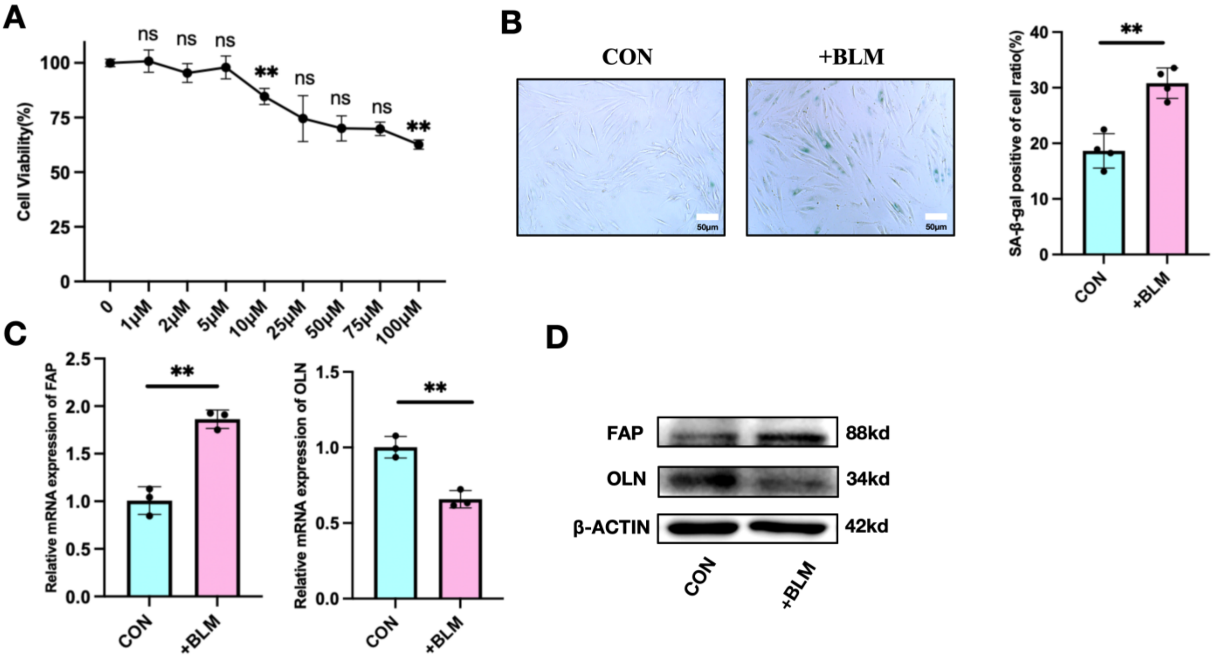

Figure S4. (A) Cell viability detected by the CCK-8 assay at different concentration of bleomycin after 24 hours of stimulation; $n = 4$. (B) Staining and quantitative analysis of SA-β-gal of H-HGF with or without bleomycin stimulation; scale bar = 50μm. (C) RT-PCR analysis quantified relative gene expression of FAP and OLN normalized to β-ACTIN of H-HGF with or without bleomycin stimulation. (D) Western blot image of FAP and OLN protein levels of H-HGF with or without bleomycin stimulation. *$P < 0.05$, **$P < 0.01$, ***$P < 0.001$, ****$P < 0.0001$.

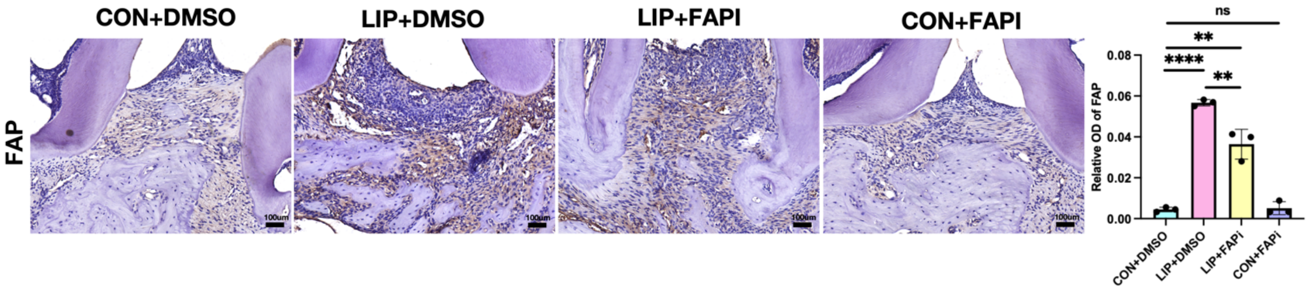

Figure S5. Representative images of IHC staining and MOD values of FAP; scale bar: 100μm; $n = 3$; *$P < 0.05$, **$P < 0.01$, ***$P < 0.001$, ****$P < 0.0001$; MOD: mean optical density.

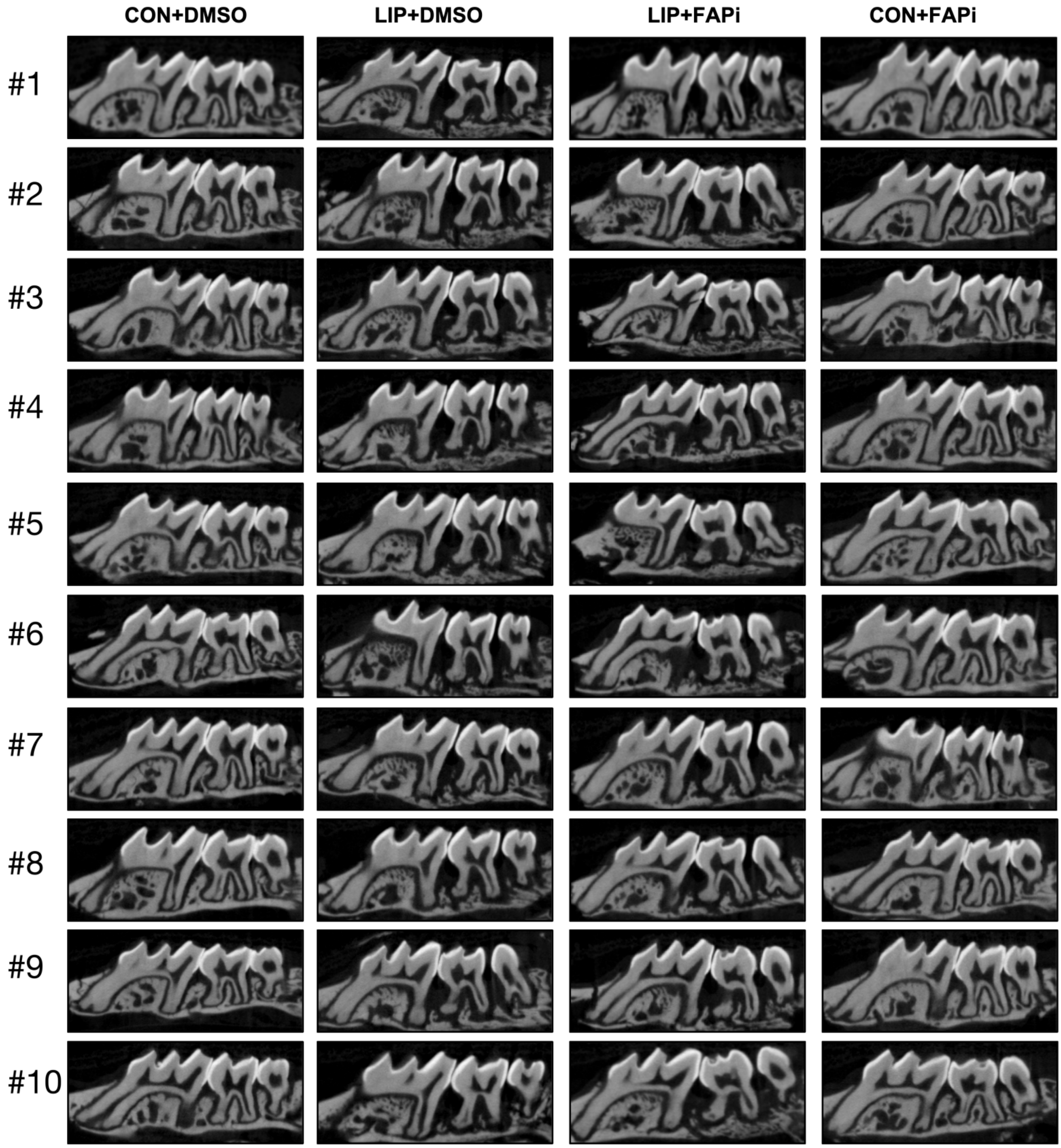

Figure S6. All the Micro-CT images from the experimental mice in Figure 5B.

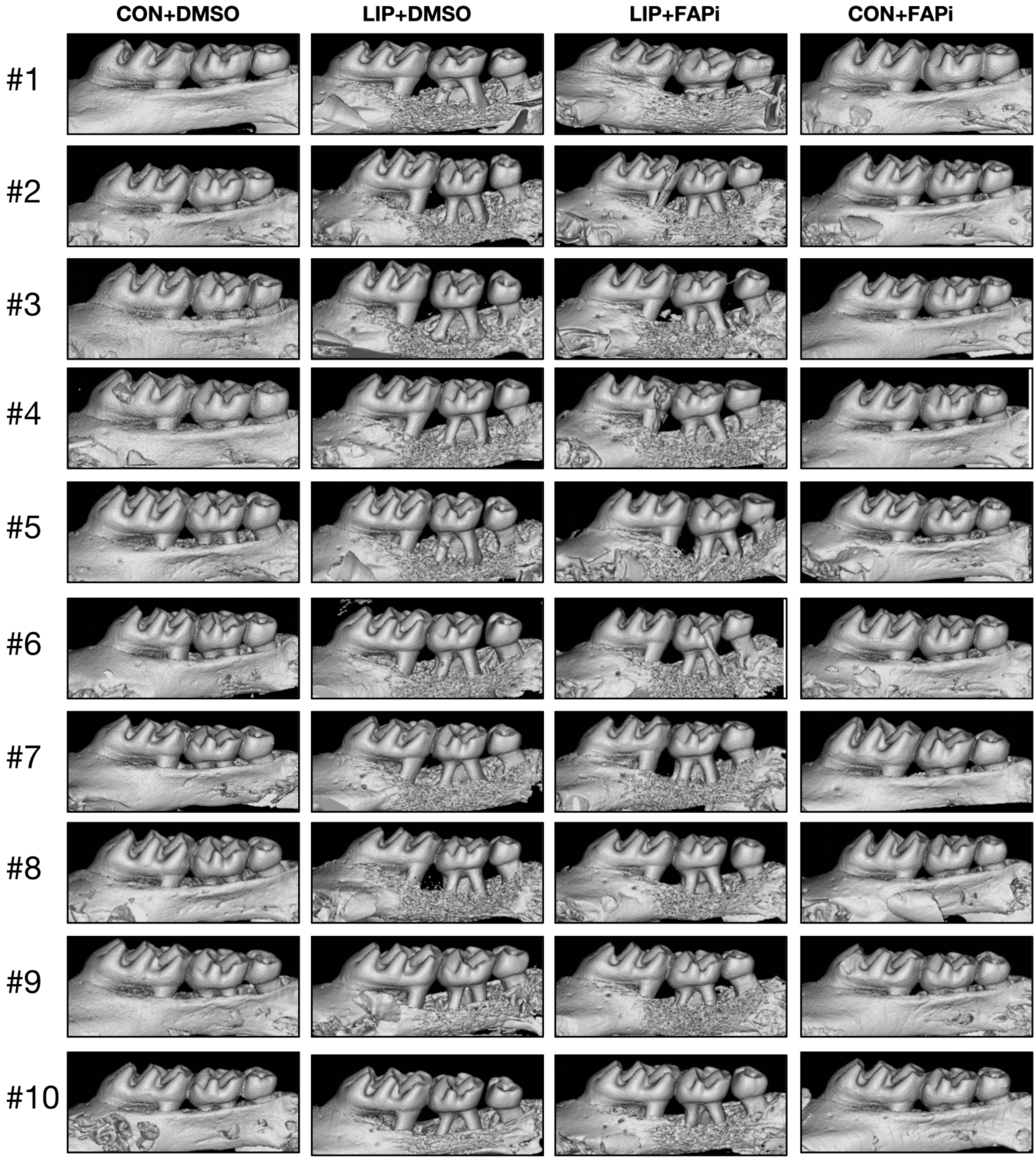

Figure S7. All the 3-Dimensional visualization of the maxilla images from the experimental mice in Figure 5C.
